# Solid Fuel Combustion and Air Pollution: Filling the Data Gap and Future Priorities

**DOI:** 10.3390/ijerph192215024

**Published:** 2022-11-15

**Authors:** Wei Du, Long Chen, Yuanchen Chen

**Affiliations:** 1 Yunnan Provincial Key Laboratory of Soil Carbon Sequestration and Pollution Control, Faculty of Environmental Science & Engineering, Kunming University of Science &Technology, Kunming 650500, China; 2 Key Laboratory of Geographic Information Science of the Ministry of Education, School of Geographic Sciences, East China Normal University, Shanghai 200241, China; 3 Key Laboratory of Microbial Technology for Industrial Pollution Control of Zhejiang Province, College of Environment, Zhejiang University of Technology, Hangzhou 310032, China

To date, some 3 billion people worldwide still rely on solid fuels (e.g., wood, coal, crop residues, animal dung, etc.) as a source of residential energy for cooking and space heating. The household air pollution (HAP) caused by pollutant emissions from solid fuel combustion is receiving increasing attention. Globally, it is estimated that more than 3.2 million premature deaths are caused by HAP associated with solid fuel use in residential homes, demonstrating an urgent need to investigate the impact of solid fuel use on human health.

In the first edition of the Special Issue “Solid Fuel Combustion and Air Pollution”, a total of eight papers were accepted, with an acceptance rate of 44.4%. The low acceptance rate illustrates the quality of the peer review and manuscripts in this **Special Issue**. When submissions were closed, many planned papers had not yet been submitted. With this in mind, the second edition of this Special issue is now open for submission. Here, we highlight the key innovations in the accepted manuscripts in the first edition, and announce the most highly valued papers that fall within the scope for the second edition.

Among the published papers in the first edition, the drivers of energy choices, the emission characteristics of solid fuel combustion, spatial and temporal variations in air pollution at different scales, and the health effects of the particle composition of solid fuel combustion were systematically discussed [1,2,3,4,5,6,7,8]. *Mperejekumana* et al. revealed that higher socio-economic levels (credit access) could increase the likelihood of choosing LPG by 22.7%, while higher levels of education can also help residents to choose cleaner residential fuels [1]. The drivers of energy choices are crucial for policy makers, but are not yet fully understood. Many factors should be taken into consideration, such as the price of various energy choices, the education level of consumers, the supply of energy, and government subsidies, which all deserve future study. The emission characteristics of solid fuel combustion, especially measurements based on real-world study, are highly valued at this stage. It has been found that coal combustion could increase the number of ultrafine particles in indoor environments, especially during the ignition period, highlighting the impact of solid fuel combustion on indoor air quality [2]. While field studies can be effective in assessing the actual impact of solid fuel use on air pollution, laboratory studies can quantify the factors affecting the emissions of various pollutants [3]. Therefore, more research into the emission characteristics of solid fuel combustion, both in the field and in the laboratory, are encouraged. Some publications focused on the factors affecting the emission inventory for urban or national air pollution [4,5,6], which also contributes to understanding the impacts of various sources, including solid fuels. A prospective cohort study looking at the health effects of biomass smoke on children found that prolonged exposure to unimproved stoves increased the risk of maternal-reported allergic asthma (odd ratios, OR = 2.42, 95% confidence of intervals, 95% CI: 1.11–5.48) and rhinitis symptoms (OR = 2.01, 95% CI: 1.13–3.58) [7]. Biomass smoke has been a concern in recent decades, not only because of its high mass concentration, but also because of its toxic composition, which binds to particles. An animal study found that the concentration of rare earth elements (REEs) in fur samples exhibited significant dose–response relationships, which highlighted the importance of REE emissions from solid fuel combustion.

The first edition of this Special Issue on solid fuel combustion and air pollution in China has achieved remarkable success, but some new research hotspots have since emerged. Therefore, we hope that the papers submitted in the second edition of this Special Issue will focus on the following topics:

1. The emission characteristics of solid fuel combustion, both in the field and in the laboratory. Target pollutants are not only limited to particulate matter, but can also include other air pollutants that have been shown to affect human health and the climate, such as amines, polycyclic aromatic hydrocarbons (PAHs) and their derivatives, heavy metals, brown carbons, persistent organic pollutants (POPs), and REEs.

2. HAP, including its pollution characteristics and toxic components. Considering that solid fuel use affects household air more significantly than ambient air and that humans spend most of their time indoors, further studies focused on this topic are urgently needed.

3. The dynamics of emissions and household air. With the development of sensor technology, it is becoming increasingly easy to evaluate the dynamic change in air pollution. Because high-resolution data can provide more information, it is possible to investigate the effects of solid fuel combustion on household air pollution and of human exposure to air pollutants.

4. The relationship between emissions and HAP. While it is well known that solid fuel combustion increases household air pollution, the relationship between the two is unclear due to a lack of quantitative description. It is hoped that future studies will be able to quantify this relationship via modeling, at both the single-household and national scale.

5. The health effects of HAP from residential combustion. Unlike the effects of ambient air pollution, the health effects of HAP are unclear. Particulate matter and gas pollutants in household air are very different from those in the ambient air, because household air is directly affected by emissions from solid combustion; hence, it is urgent to study the effects of household air pollution on human health.

In summary, HAP associated with solid fuel combustion is of concern, but there is currently insufficient awareness and limited information. We encourage the submission of high-quality papers to this second edition of the **Special Issue** “Solid Fuel Combustion and Air Pollution”, aiming to provide a first-hand database for academics and policy makers.
